# Gender differences in aggression of borderline personality disorder

**DOI:** 10.1186/s40479-015-0028-7

**Published:** 2015-04-09

**Authors:** Falk Mancke, Katja Bertsch, Sabine C Herpertz

**Affiliations:** Department of General Psychiatry, University of Heidelberg, Heidelberg, Germany

**Keywords:** Borderline personality disorder, Aggression, Gender differences, Neurobiology

## Abstract

Aggression is a core feature of borderline personality disorder (BPD). Well-replicated results from the general population indicate that men engage in aggression more frequently than women. This article addresses the question of whether gender also influences aggression in BPD, and whether the neurobiological mechanisms underlying aggressive behavior differ between male and female BPD patients. Data show that most self-reports, interviews and behavioral tasks investigating samples of BPD patients do not find enhanced aggressiveness in male patients, suggesting that BPD attenuates rather than aggravates gender differences usually present in the general population. Neurobiological studies comparing BPD patients with gender-matched healthy controls, however, reveal a number of interesting gender differences: On the one hand, there are well-replicated findings of reduced amygdala and hippocampal gray matter volumes in female BPD patients, while these findings are not shared by male patients with BPD. On the other hand, only male BPD patients exhibit reduced gray matter volume of the anterior cingulate cortex, increased gray matter volume of the putamen, reduced striatal activity during an aggression task, and a more pronounced deficit in central serotonergic responsivity. These neurobiological findings point to a particular importance of impulsivity for the aggression of male BPD patients. Limitations include the need to control for confounding influences of comorbidities, particularly as male BPD patients have been consistently found to show higher percentages of aggression-predisposing comorbid disorders, such as antisocial personality disorder, than female BPD patients. In the future, studies which include systematic comparisons between females and males are warranted in order to disentangle gender differences in aggression of BPD patients with the aim of establishing gender-sensitive treatments where needed.

**Electronic supplementary material:**

The online version of this article (doi:10.1186/s40479-015-0028-7) contains supplementary material, which is available to authorized users.

## Background

Aggression may be defined as any behavior directed toward another individual with the intention to cause harm [1]. Dysregulated anger and its behavioral manifestations such as physical fights are among the defining criteria of borderline personality disorder (BPD) [2]. Several findings emphasize the high prevalence of aggression in BPD: 73% of BPD patients have engaged in aggressive behavior over the course of a year [3], 58% are “occasionally or often” involved in physical fights, and 25% have used a weapon against others ([2], p.154). BPD patients constitute a major proportion of prison inmates, with prevalence rates of 30% [4]. Increased aggression in BPD has been found using both categorical [5-7] and dimensional measurements [8-11] of BPD symptomatology, and may thus be regarded as a core feature of the disorder [12,13].

In the general population, men engage in aggression more frequently than women, with effect sizes ranging between 0.14 – 0.84 (weighted *d*) depending on the method of data collection and the form of aggressive behavior: The effect size is stronger in real-world settings and varies as a function of the seriousness of the aggressive act, i.e., the more dangerous the behavior, the stronger the male preponderance [14,15]. However, it remains unclear whether and how gender influences aggression in BPD. In the current article, we will first analyze whether female and male BPD patients differ in their propensity to behave aggressively by reviewing data from self-reports, interviews and behavioral tasks. Second, we will analyze gender differences in the neurobiological underpinnings of BPD patients’ aggression.

## Review

### Self-reports, interviews and behavioral tasks

Self-report measures, such as the Buss-Durkee Hostility Inventory [16] and its revised form, the Buss and Perry Aggression Questionnaire [17], have been used to assess trait aggressiveness by asking about aggressive behaviors and aggression-predisposing emotional experiences, such as intense anger. In the context of intimate partner aggression, the most frequently used questionnaire is the Revised Conflict Tactics Scale [18]. Semi-structured interviews that measure aggression have focused on the quantification of observable and direct aggressive behaviors, e.g., shouting and fighting. These interviews differ in terms of the length of the investigated timespan, measuring aggression either across the lifetime [the Brown-Goodwin Lifetime History of Aggression interview [19] and its revised form, the Life History of Aggression interview [20]], or in the last two weeks [the Modified Overt Aggression Scale [21]]. Semi-structured interviews based on classifications of mental disorders, like the Structured Clinical Interview for DSM Disorders [22], do not directly measure aggression, but can nevertheless provide valuable insights as they assess the frequency of aggression-predisposing-disorders such as antisocial personality disorder (ASPD). The most frequently used behavioral task to measure aggressive behavior in BPD is the Point Subtraction Aggression Paradigm (PSAP) [23,24]. The PSAP uses interpersonal provocation, i.e., the subtraction by a fictitious opponent of “points” worth money that the participant has accumulated during testing. Ignoring the provocation and thereby accumulating points is considered as the “monetary-reinforced response”, while reacting in a retaliatory manner by subtracting points from the fictitious opponent – without garnering anything – is considered as the “aggressive response” and is used as the measure of aggression (see, e.g., [25], for a detailed description of the PSAP). The following section reviews gender differences in BPD from studies using these instruments. Table 1 provides a detailed description of the cited studies, including sample characteristics, methodology and key findings.

**Table 1:** **Studies investigating aggression in ♀ and ♂ BPD patients using self-reports, interviews and behavioral tasks**

**First author**	**Year**	**Sample**	**Methodology**	**Key findings**
Banzhaff et al.	2012	170 BPD patients (114 ♀, 56 ♂)	BPD: Structured Clinical Interview for DSM-IV for personality disorders (SCID-II)	♂ BPD patients had higher prevalence of ASPD than ♀ BPD patients (32.14 % vs. 10.53 %, p < .001). ♂ BPD patients had higher score in “Dissocial behavior” of the DAPP-PQ than ♀ BPD patients (p < .004).
			Aggression: Subscale Dissocial behavior of the Dimensional Assessment of Personality Profile Basic questionnaire (DAPP-BQ)	
Barrachina et al.	2011	484 BPD patients (402 ♀, 82 ♂)	BPD: SCID II, Diagnostic Interview for Borderlines-Revised (DIB-R)	♂ BPD patients had higher prevalence of ASPD than ♀ BPD patients (22% vs. 8.2 %, p < .008).
Black et al.	2007	220 offenders newly committed to prison (198 ♂, 22 ♀)	BPD: Structured Interview for DSM-IV Personality (SIDP-IV)	65 offenders (29.5%) met criteria for BPD. More women (54.5%) than men (26.8%) met criteria for BPD (p = .007).
Brambilla	2004	10 BPD patients ((6 ♀, 4 ♂), 20 HC (gender ratio: n/a)	BPD: IPDE	BPD showed higher volume of the putamen (p = .002) compared with HC.
			Neuroimaging: stMRI, manual tracing	
Costa et al.	2008	130 intimate aggressive ♂, 48 non aggressive ♂	BPD: Millon Clinical Multiaxial Inventory-III	Jealousy correlated positively with BPD symptomatology (r = .13, p < .05).
			Intimate partner aggression: Revised conflict tactics scale (CTS2), General Violence Questionnaire	
Grant et al.	2008	2004 BPD patients (gender distribution not mentioned)	BPD: Alcohol Use Disorder and Associated Disabilities Interview Schedule DSM-IV Version	♂ BPD patients had higher prevalence of ASPD than ♀ BPD patients (19.4% vs. 9 %, p < .001).
Grilo et al.	2002	100 BPD patients (69 ♀, 31 ♂)	BPD: Diagnostic Interview for DSM-IV Personality Disorders (DIPD-IV)	♂ BPD patients had higher prevalence of ASPD than ♀ BPD patients (48% vs. 0 %).
Hines et al.	2008	14,154 university-students (10100 ♀, 4054 ♂)	BPD: Personal and Relationships Profile	No BPD x gender interaction for physical, psychological and sexual intimate partner aggression (p > .05 for all contrasts).
			Intimate partner aggression: CTS2	
Holtzworth et al.	2000	102 intimate aggressive ♂, 62 non aggressive ♂	BPD: Millon Clinical Multiaxial Inventory-III	Identified four clusters of violent men, among them the so-called borderline-dysphoric men characterized by high measures on dependency, jealousy, impulsivity and hostility towards women.
			Intimate partner aggression: CTS2, Generality of Violence Questionnaire	
Johnson et al.	2003	240 BPD patients (175 ♀, 65 ♂)	BPD: DIPD-IV	♂ BPD patients had higher prevalence of ASPD than ♀ BPD patients (29.7% vs. 10.3 %, p < .0001).
McCloskey et al.	2009	127 BPD patients (69 ♀, 58 ♂) of whom 40 with comorbid ASPD, clinical control group consisting of 122 patients with a non cluster-B personality disorder (57 ♀, 65 ♂) and 112 HC (55 ♀, 57 ♂)	BPD: Structured Interview for DSM-IV Personality (SIDP-IV)	No group x gender interaction in the AQ (Wilks F <1).
				No difference between ♂ and ♀ BPD patients in the post-hoc analysis of the LHA (p < .01, based on personal communication). ♀ BPD patients were more self-aggressive than the ♂ BPD patients (p < .01)
			Aggression: Life History of Aggression (LHA), Aggression Questionnaire (AQ), Point Subtraction Aggression Paradigm (PSAP)	
				No effect of gender or gender x group interaction in the PSAP (Wilks F < 1).
McCormick et al.	2007	163 BPD patients (138 ♀, 25 ♂)	BPD: SIDP-IV	♂ BPD patients had higher prevalence of ASPD than ♀ BPD patients (40% vs. 21 %, p < .03).
Newhill et al.	2009	220 BPD patients (116 ♀, 104 ♂)	BPD: Structured Interview for DSM-III-R Personality	No gender difference in aggression of BPD patients (p = .342)
			Aggression: arrest records, collateral reports, patients report using behaviors adapted from the CTS2	
Prehn et al.	2013	15 ♂ BPD-ASPD, 17 ♂ HC	BPD: IPDE	♂ BPD-ASPD displayed increased amygdala activity exclusively in response to high, but not neutral and low emotional stimuli when compared to ♂HC.
			Neuroimaging: fMRI during presentation of emotional & neutral pictures	
Ross et al.	2009	124 intimate aggressive ♂ (7 BPD patients, 16 BPD-ASPD, 18 ASPD patients and 83 subjects without a personality disorder)	BPD: SCID-II	♂ BPD-ASPD were more likely than ASPD-patients (p < .01) and subjects without a personality disorder (p < .01) to react aggressively upon women’s displays of stress.
			Intimate partner aggression: CTS2	
Scott et al.,	2014	75 psychiatric outpatients and 75 community residents (98 ♀, 52 ♂)	BPD: dimensional score using a Diagnostic and Statistical Manual of Mental Disorders checklist and the Structured Interview for DSM-IV Personality	Gender did not influence the prediction of BPD on aggression.
			Aggression: Revised Conflict Tactics Scale	
Silberschmidt et al.	2015	770 BPD (559 ♀, 211 ♂)	BPD: DIPD-IV	No gender difference in aggression of BPD patients (p = .193). ♀ BPD patients showed enhanced hostility than the ♂ BPD (p = .011).
			Aggression: OAS-M	
			Hostility: The Symptom Checklist 90 Revised	
Tadic et al.	2009	159 (110 ♀, 49 ♂)	BPD: SCID-II	♂ BPD patients had higher prevalence of ASPD and higher prevalence of the criterion intensive anger (73.5% vs 49.1%, p < .001) than ♀ BPD patients (57.1% vs. 25.51%, p < .001).
Weinstein et al.	2012	847 late middle-age (55-64) adults (347 ♀, 500 ♂) from a community sample	BPD: SIDP-IV, Multi-Source Assessment of Personality Pathology (MAPP) fulfilled by the participant and an informant	In ♀, but not ♂, subjects intimate partner aggression was related to BPD traits (regression-coefficients: 37.5 for the SIDP-IV, 10.2 for the self-MAPP and 8.9 for the informant-MAPP).
			Intimate partner aggression: CTS2	
Zanarini et al.	1998	379 BPD patients (296 ♀, 83 ♂)	BPD: Diagnostic Interview for DSM-III-R Personality Disorders, DIB-R	♂ BPD patients had higher prevalence of ASPD than ♀ BPD patients (48 % vs. 16 %, p < .00001).
Zlotnick et al.	2003	149 BPD patients (104 ♀, 45 ♂)	BPD: SIDP-IV	♂ BPD patients had higher prevalence of ASPD than ♀ BPD patients (38.6% vs. 11.4 %, p < .001).

The studies are listed in alphabetical order based on the author’s first name.

*Abbreviations*: AQ: Aggression Questionnaire, BG-LHA: Brown-Goodwin Lifetime History of aggression, BDHI: Buss-Durkee Hostility Inventory, DIB-R: Diagnostic Interview for Borderlines- Revised, CTS2: Revised conflict tactics scale, DAPP-BQ: Dimensional Assessment of Personality Profile Basic questionnaire, IPDE: International Personality Disorders Examination, LHA: Life History of Aggression, MAPP: Multi-Source Assessment of Personality Pathology, m SCID-II: Structured Clinical Interview for DSM-IV Axis II Personality Disorders, OAS-M: modified Overt Aggression Scale SIDP-IV: Structured Interview for DSM-IV Personality.

Using the Brown-Goodwin Lifetime History to measure aggression in BPD patients, some [26,27] but not all [28] studies reported enhanced aggression in the male compared to female patients. Data from studies applying the Buss-Durkee Hostility Inventory are inconsistent: One study using this instrument [26] found more aggression in male than in female BPD patients, while another study did not [29]. No differences between male and female BPD patients were found in studies using the Buss and Perry Aggression Questionnaire [6], the Modified Overt Aggression Scale [29], and the Life History of Aggression [4, personal communication]. The Modified Overt Aggression Scale was also applied in a study by Silberschmidt and coworkers [30]. This study is remarkable for its large sample size particularly with regard to male subjects (559 female and 211 male BPD patients) [30]. However, despite adequate statistical power, no difference between male and female BPD patients emerged. Instead, this study revealed enhanced hostility of female compared to male patients. More recently, Scott and colleagues [31] performed a prospective study in a mixed clinical and community sample and used the Revised Conflict Tactics Scale as a measure for aggressiveness. They also demonstrated that the relationship between BPD traits and aggression was not influenced by gender. This is consistent to the findings of another longitudinal study in which no gender differences were found in a threefold aggression measurement consisting of arrest records, collateral informants, and patient self-reports [3].

When evaluating these results, the sample characteristics of the respective studies need to be taken into account: All of the patients in the study of New et al. [29] suffered from comorbid intermittent-explosive disorder. Additionally, roughly one third of the BPD patients in the cited studies (specifically: 26% in [27], 24.2% in [28], 31.5% in [6], 26% in [29], and 33% in [3]) fulfilled the criteria of ASPD. Although gender ratios in comorbid ASPD are not consistently reported, this high amount of comorbidities with other aggression-predisposing disorders limits the ability to draw BPD-specific conclusions and warrants caution when interpreting the results.

A considerable amount of research has analyzed the impact of BPD symptoms on intimate partner violence. Holtzworth-Munroe and Stuart [27,28], for instance, identified the so-called “borderline-dysphoric batterer”, characterized by moodiness, fear of abandonment, and insecure attachment patterns, which was related to intimate partner aggression by men against women. In a related vein, Tragesser and Benfield [34] demonstrated that the mate retention tactic of emotional manipulation (e.g., telling one’s partner that you are dependent on him or her, that you need him or her) – a tactic which predicted intimate partner aggression in previous research [34] – was positively associated with BPD traits among men but not among women.

Research on intimate partner violence has traditionally focused on male subjects. However, recent cross-gender analyses also point towards an influence of BPD on female-to-male intimate partner violence. In a study with more than 14,000 male and female students BPD traits were associated with intimate partner violence (measured with the Revised Conflict Tactics Scale) not only in male, but also in female students [8]. Using the same instrument, another study found a positive relationship between BPD traits and intimate partner aggression in female, but not in male participants of a late-middle-aged community sample [35]. The latter finding might have been due to the form of aggressive behavior examined: It consisted mostly of verbal aggression, e.g., shouting at the partner, and this form of aggression has previously been shown to be associated with a reduced gender difference in the male direction in the general population [15].

Although self-aggressive behavior has often been considered as distinct from aggression directed towards other individuals, aggression measurements, such as the Life History of Aggression interview, frequently include subscales for self-aggressive behavior. Female BPD patients have been found to report more self-aggressive acts than their male counterparts in the Life History of Aggression interview [4, personal communication]. Similar to the female-only association between BPD traits and verbal aggression reported above, this finding marks the importance of considering the specific characteristics of the studied behavior when evaluating gender differences in aggression.

Several studies using semi-structured interviews investigated whether male and female BPD patients differ in the prevalence of comorbid disorders that predispose them to aggression, such as ASPD. Male BPD patients were more frequently found to meet the diagnostic criteria of ASPD than female patients, irrespective of whether symptomatology was assessed dimensionally [36] or categorically [37-44]. On the level of DSM-IV criteria, one study [40] found that male compared to female BPD patients more often fulfilled the criterion of intensive anger, while this was not found in another study [39].

Studies have repeatedly found more aggressive responses on the PSAP in patients with BPD compared to healthy individuals [6,29,45]. However, male and female BPD patients did not differ in their aggressive responses [6,29]. Instead, a gender difference was found in what is considered to be the “monetary-reinforced response” (for details, see above): Male controls chose this response more often, thereby garnering more points at the end of the task, than the male BPD patients. The opposite pattern was observed for the women: Female BPD patients chose the “monetary-reinforced response” more frequently than female controls, which – although counterintuitive – suggests enhanced behavioral control of female BPD patients compared to female controls. However, both studies using the PSAP did not report any gender effect in healthy controls; this result has to be interpreted in the context of the type of provocation used by the PSAP. It uses interpersonal frustration, which has been associated with a smaller gender difference in aggression in healthy samples [46].

Taken together, results from self-report and interview-based measurements and behavioral tasks in BPD patients show a different pattern than in the general population. In the general population, men have been found to engage in more aggression than women, whereas in BPD, most studies did not find such a gender difference in aggression. Findings reporting more verbal aggression in female subjects scoring high on BPD traits than their male counterparts emphasize the importance of differentiating between specific forms of aggression. Male BPD patients have been consistently found to show higher percentages of aggression-predisposing disorders such as ASPD. Research on intimate partner aggression has concentrated on male-to-female aggression and indicates a predisposing effect of BPD on intimate partner aggression, which might stem from different mate retention tactics. However, as the number of studies is limited, and as results are often confounded by comorbidity and grouping together of different forms of aggression, further research is urgently needed.

### Neurobiology

Aggressive behavior is understood to result from biological as well as environmental vulnerabilities (see, e.g., [47] for a review). Environmental risk factors are numerous and include maltreatment, smoking during pregnancy, divorce, peer deviance, parental psychopathology, social disadvantage, and coercive discipline [48]. The latter is thought to establish reinforcement contingencies that shape and maintain deviant and aggressive behaviors [49,50]. Theories on the biological underpinnings of aggression in BPD propose a brain circuitry implicating predominantly prefrontal and limbic structures. More specifically, a model has been proposed in which prefrontal regions, especially the orbital frontal cortex and the anterior cingulate cortex, fail to control enhanced reactivity of limbic regions such as the amygdala [see, e.g., 43, for a review]. The insufficiency of prefrontal regions in regulating limbic hyperactivity has been consistently related to deficiency of the prefrontal serotonergic system [52]. This prefrontal-limbic imbalance was shown to be associated with BPD patients’ propensity to perceive emotionally challenging stimuli as provocative and threatening, and thus favoring anger and ultimately aggressive behavior [51,53]. Brain areas additionally modulating the aggressive response include hippocampal [54] and hypothalamic structures [54,55].

The interaction between environmental and biological factors is rather synergistic than additive (see, e. g., [56] for a review). For instance, in the landmark paper of Caspi et al. [57], the vulnerable genotype alone explained less than 1 % of the variance in antisocial behaviors, including aggression. However, in combination with maltreatment its fraction of explained variance rose to 65 %. The moderation of gene x environment interactions by gender may therefore be a highly interesting topic for future research.

In the following paragraph, we will evaluate gender differences in the neurobiological systems underlying BPD patients’ aggression. Table 2 provides a detailed description of the cited studies, including sample characteristics, methodology, and key findings.

**Table 2:** **Studies investigating aggression in ♀ and/or ♂ BPD patients using neurobiological methods**

**First Author**	**Year**	**Sample**	**Methodology**	**Key findings**
Bertsch et al.	2013	39 ♂ BPD patients with comorbid ASPD (BPD-ASPD), 14 HC	BPD: International Personaliy Disorder Examination (IPDE)	♂ BPD-ASPD patients displayed volume reduction in the left frontal pole, left orbital frontal cortex and right ventromedial prefrontal cortex compared to ♂ HC (all p < .05, ROI-analysis).
			Neuroimaging: strucutural magnetic resonance imaging (stMRI), voxel based morphometry (VBM)	
Brambilla	2004	10 BPD patients ((6 ♀, 4 ♂), 20 HC (gender ratio: n/a)	BPD: IPDE	BPD showed higher volume of the putamen (p = .002) compared with HC.
			Neuroimaging: stMRI, manual tracing	
Coccaro et al.	2007	31 ♂ personality-disorders subjects, including 4 BPD patients	BPD: SCID-II	Testosterone CSF concentration of ♂ personality disordered patients, including BPD, is not correlated with aggression (p = .34).
			Aggression: Life History of Aggression (LHA), criteria for Intermittend Explosive Disorder	
			Neurochemistry: Cerebrospinal fluid (CSF) concentration of testosterone	
Coccaro et al.	1998	26 personality-disordered subjects, including 7 BPD patients (8 ♀, 18 ♂)	BPD: According to DSM-IV criteria	In the personality-disordered patients, including BPD, vasopressin CSF concentration was positively correlated with aggression (r = .41, p = .04), which was stronger in ♂ (r = .65) than in ♀ subjects (r = .27).
			Aggression: LHA	
			Neurochemistry: CSF concentration of vasopressin	
Hazlett et al.,	2005	50 BPD patients (23 ♀, 27 ♂), 50 HC (20 ♀, 30 ♂)	BPD: Structured Interview for DSM-III-R Personality	BPD showed reduced gray matter and more white matter volume in BA 24 and 31 of the cingulate compared with HC (all p < .01,ROI analysis).
			Neuroimaging: stMRI, manual tracing	
Hollander et al.	1994	12 BPD patients (8 ♀, 4 ♂), 15 HC (3 ♀, 12 ♂)	BPD: SCID-II	Diminished serotonergic responsivity in ♂, but not ♀ BPD patients compared to gender-matched HC (p = .010)
			Neurochemistry: Serotonergic responsivity via m-chlorophenylpiperazine (m-CPP)	
Martial et al.	1997	5 ♀ BPD patients	BPD: DIB-R	No diminished serotonergic responsivity of ♀ BPD patients.
			Neurochemistry: Serotonergic responsivity via d-fenfluramine (FEN)	
Minzenberg	2008	12 BPD-patients (5 ♀, 7 ♂), 12 HC (6 ♀, 6 ♂)	BPD: Structured Interview for DSM-IV Personality	BPD had reduced gray matter volume in the anterior cingulate (BA 24/32) compared to HC (p < .003-007, ROI analysis).
			Neuroimaging: stMRI, VBM	
New/Perez-Rodriguez et al.	2009/ 2012	38 BPD with comorbid intermittent-explosive-disorder (16 ♀, 22 ♂), 36 HC (18 ♀, 18 ♂)	BPD: SCID-II	No group x gender interaction in the OAS-M, BDHI and the AQ. ♀ BPD patients chose the “right” answer in the PSAP more often than ♀ HC. ♂ BPD patients chose the “right” answer in the PSAP less frequently than ♂ HC (p < 0.005). ♂ BPD displayed reduced glucose metabolism rate in the striatum when performing the PSAP compared to ♀ BPD and HC of both gender (p < .01). No gender differences in prefrontal and amygdala regions.
			Aggression: modified Overt Aggression Scale (OAS-M),	
			Buss-Durkee Hostility Inventory (BDHI), AQ, PSAP	
			Neuroimaging: PET while performing PSAP	
Niedtfeld et al.	2010	23 ♀ BPD patients, 26 ♀ HC	BPD: International Personality Disorders Examination (IPDE)	♀ BPD patients displayed increased amygdala reactivity in response to neutral and negative emotional stimuli when compared to ♀ HC.
			Neuroimaging: functional magnetic resonance (fMRI) during presentation of emotional & neutral pictures	
Prehn et al.	2013	15 ♂ BPD-ASPD, 17 ♂ HC	BPD: IPDE	♂ BPD-ASPD displayed increased amygdala activity exclusively in response to high, but not neutral and low emotional stimuli when compared to ♂HC.
			Neuroimaging: fMRI during presentation of emotional & neutral pictures	
Rinne et al.	2000	12 ♀ BPD patients, 9 ♀ HC	BPD: Structured Interview for DSM III-R Personality	♀ BPD patients displayed diminished serotonergic responsivity than ♀ HC (p < .05).
			Disorders	
			Neurochemistry: Serotonergic responsivity via m-CPP	
Soloff et al.	2003	64 BPD patients (44 ♀, 20 ♂), 57 HC (21 ♀, 36 ♂)	BPD: IPDE	♂ BPD patients showed higher scores in the BDHI (p = 0.03) and the BG-LHA (p = .002) than the ♀ BPD patients. ♂, but not ♀, BPD patients, had significantly lower delta-prl (p < .02), peak-prl (p < .001) and AUC-prl (p < .003) compared to gender-matched HC. In ♂, but not ♀, BPD patients serotonergic responsivity was inversely correlated with scores of the LHA (p < .05)
			Aggression: BDHI, Brown-Goodwin Lifetime History of aggression (BG-LHA)	
			Neurochemistry: Serotonergic responsivity via FEN	
Soloff et al.	2005	22 BPD patients (15 ♀, 7 ♂), 24 HC (14 ♀, 10 ♂)	BPD: IPDE, DIB-R	♀, but not ♂, BPD patients displayed reduced glucose metabolism rate in the prefrontal cortex during baseline condition compared to gender-matched HC (p < .05). When tested for serotonergic responsivity ♂ BPD patients, but not ♀ BPD patients, showed decreased glucose metabolization in the lt. temporal and frontal lobe (p < .05)
			Aggression: BG-LHA	
			Neurochemistry: serotonergic responsivity via FEN Neuroimaging: PET during baseline and serotonergic responsivity condition	
Soloff et al.	2008	34 BPD patients (22 ♀, 12 ♂); among them 2 ♀ BPD-ASPD and seven ♂ BPD-ASPD, 30 HC (19 ♀, 11 ♂)	BPD: IPDE	♂ BPD patients had higher scores in the BG-LHA than ♀ BPD patients (p = .03). ♀, but not ♂, BPD patients showed a gray volume reduction the amygdala, hippocampus bilaterally compared to gender-matched HC (all p_fwe_ < .05). ♂, but not ♀, BPD patients showed a gray matter volume reduction in the anterior cingulate cortex (p_cluster_ = .001) and a gray matter volume increase in the right putamen (p_cluster_ = .024) when compared to gender-matched HC.
			Aggression: BG-LHA	
			Neuroimaging: stMRI, VBM	
Völlm et al.	2009	7 ♂ BPD patients, 6 ♂ HC	BPD: SCID-II	♂ BPD patients showed gray matter volume reduction in the medial, middle and superior frontal gyrus, bilaterally, and the left orbitofrontal cortex and right anterior cingulate cortex compared to ♂ HC (all at least p_uncorr._ < .001).
			Neuroimaging: stMRI, VBM	

The studies are listed in alphabetical order based on the author’s first name.

*Abbreviations*:: BG-LHA: Brown-Goodwin Lifetime History of aggression, BPD-ASPD: BPD patients with comorbid ASPD, BDHI: Buss-Durkee Hostility Inventory, CSF: cerebrospinal fluid, DIB-R: Diagnostic Interview for Borderlines- Revised, International Personality Disorders Examination, fMRI: functional magnetic resonance imaging, LHA: Life History of Aggression,, m-CPP: m-chlorophenylpiperazine, PSAP: Point Subtraction Aggression Paradigm SCID-II: Structured Clinical Interview for DSM-IV Axis II Personality Disorders, OAS-M: modified Overt Aggression Scale SIDP-IV: Structured Interview for DSM-IV Personality, stMRI: structural magentic resonance imaging, PET: positron emission tomography, ROI: region of interest, VBM: voxel based morphometry.

BPD patients were found to show gender differences in brain volume: When compared to gender-matched healthy controls, female but not male patients displayed reduced gray matter volumes in the amygdala and hippocampus, while male but not female BPD patients showed reduced volume in the anterior cingulate cortex as well as increased volume in the right putamen [27]. This study did not, however, report a comparison between men and women with BPD. Other mixed-gender studies that included a notably large number of male BPD patients – but which did not perform direct gender comparisons – also reported reduced volume in the anterior cingulate cortex [50 with 54 % male BPD patients, 51 with 58 % male BPD patients] and increased volume in the putamen [52 with 40 % male BPD patients], which provides tentative support for the male-only findings in the study by Soloff et al. [27].

Studies investigating only male BPD patients compared to healthy men revealed volume reductions in the orbital frontal cortex and the ventromedial prefrontal cortex [61] as well as in the superior, medial and middle frontal gyrus [62]. Again, comorbidities need to be considered here: 58.3% of the male BPD patients but only 9.1% of the female BPD patients in the study by Soloff et al. [27], and all the participants in the work by Bertsch et al. [61], were diagnosed with ASPD, which limits the ability to interpret BPD-specific effects.

Recently, it was found that while performing the PSAP, male BPD patients with comorbid intermittent explosive disorder had a significantly lower glucose metabolism rate in the striatum (a subcortical brain structure consisting of the caudate nucleus and the putamen) than female patients [63]. No difference was found between female patients and healthy women. Notably, to date, this is one of the few neuroimaging studies to have allowed the direct comparison of male with female BPD patients. The striatum is closely connected with prefrontal areas [64] and one of its suggested roles is to recognize the situational context the organism is in [65,66]. Reduced striatal activity in male BPD patients could therefore be associated with inadequate evaluation of the situational context, which may impair inhibitory processes of prefrontal areas and might ultimately facilitate aggressive behavior. This functional neuroimaging finding shown only in male BPD patients parallels the above-mentioned structural neuroimaging alteration of the putamen. Future studies could pursue the highly interesting question of whether striatal brain structures may be a primary correlate of gender differences in aggression of BPD patients.

Interestingly, in a previous analysis of the same data by Perez-Rodriguez and colleagues [63], no gender differences in brain metabolism were found in prefrontal and amygdala areas while participants were performing the PSAP [29]. This is of interest since a positron emission tomography study by Soloff et al. [67] reported prefrontal hypometabolism during baseline condition for female but not male BPD patients when a gender-matched analysis was performed.

Functional neuroimaging studies that analyzed only male BPD patients with comorbid ASPD found increased amygdala activity exclusively in response to high, but not to neutral and low emotionally salient stimuli [68]. This is in contrast to female BPD patients, who also displayed enhanced amygdala reactivity to neutral stimuli compared to healthy women [69]. Both Prehn et al. [68] and Niedtfeld et al. [69] used stimulation material from the International Affective Picture System [70]. However, comparability between the two studies is limited, as Prehn et al. [68] presented the pictures as task-irrelevant background distractors in a working memory task, while the pictures were task-relevant and therefore in the participants’ attentional focus in the study by Niedtfeld et al. [69]. Nevertheless, these results raise the question of different amygdala activity in male versus female BPD patients, which is of particular interest since gender-specific functional [71] and structural [72] differences in the amygdala have been reported in healthy samples.

Neurochemically, the central serotonin system seems to differ substantially between male and female BPD patients. Using d-fenfluramine or m-Chlorophenylpiperazine to test the responsivity of the serotonin system, compared to gender-matched healthy volunteers, only male but not female BPD patients showed a diminished serotonergic response in most [26,73,74] but not all [75] studies. Correlational analyses further showed that only the male patients demonstrated an inverse relationship between measures of aggression (the Buss-Durkee Hostility Inventory) and serotonin responsivity [26]. Additional positron emission tomography in response to d-fenfluramine revealed that male but not female BPD patients displayed decreased glucose uptake relative to gender-matched controls, which was mainly located in the temporal lobe [67]. However, the ability to draw inferences from this study is limited, as it included more than twice as many female as male BPD patients.

Interestingly, a recent study found increased serotonin-2A receptor binding in female BPD patients compared to gender-matched controls and male BPD patients [28], which implies diminished serotonergic agonism of the female patients. Furthermore, binding potentials predicted aggression only in the female patients.

Serotonergic x environment interactions may additionally be moderated by gender. For instance, the association between the short allele of the serotonin transporter gene and laboratory measured aggression was more prominent in men than in women [76]. Contrary to this, serotonergic vulnerabilities may primarily lead to self-injurious behavior in women as shown by Crowell and coworkers [77]. These authors investigated the effect of peripheral serotonin and mother-daughter conflict on self-injurious behavior. Taken by themselves, peripheral serotonin and conflict had only limited effect, whereas their interaction explained 64% of the variance in self-injurious behavior [77]. These results – though not performed with BPD patients – illustrate the complexity of the interplay between the serotonergic system, gender and aggression.

Other neurochemical systems might additionally play a role in the differential regulation of aggression between male and female BPD patients. In a sample of personality-disordered subjects, including BPD patients, a positive correlation was found between the concentration of vasopressin in the cerebrospinal fluid and aggression, which was stronger in male than in female subjects [78]. Again, due to the small number of BPD patients in this study, interpretability is limited.

In the general population, a weak positive association between concentrations of testosterone in different body fluids (mostly blood or saliva) and antisocial, dominant, or competitive behaviors has been suggested [79]. Testosterone was also found to modulate aggressive behavior, although the direction of the association is less clear [80,81]. In a sample of male patients with various personality disorders, including BPD patients, no association between cerebrospinal fluid concentrations of testosterone and aggression was found [82]. Again, the small proportion of BPD patients in this study (4 out of 31) limits the ability to draw BPD-specific conclusions. In line with no effect of testosterone on aggression in BPD patients, one study with female BPD patients did not find an association between free serum testosterone levels and measures of aggression [83]. Plasma concentrations of oxytocin were negatively associated with trait aggressiveness in a sample of female BPD patients [84]; so far, findings from male BPD patients in this respect are lacking.

In sum, results from neurobiological studies suggest a different pattern of alterations between male and female BPD patients. Male but not female BPD patients when compared to gender-matched healthy volunteers exhibited reduced gray matter volume of the anterior cingulate cortex, increased gray matter volume of the putamen, reduced striatal activity during an aggression task, and a more pronounced deficit in central serotonergic responsivity. Specific alterations found in female BPD patients are rare. The few findings that exist raise the question of whether dysfunctions in the amygdala are more pronounced in female than in male BPD patients.

## Conclusions

This article addressed the question of gender differences in BPD patients’ aggressive behavior. In the general population, men show enhanced aggression compared with women. By contrast, most results from self-reports, interviews, and behavioral tasks do not indicate any gender differences in the patients’ aggressiveness. Taken together, these results suggest that BPD attenuates rather than aggravates the gender difference in aggression normally present in the general population.

This article also reviewed gender differences in the neurobiological underpinnings of BPD patients’ aggression. Conclusions from these findings require a conceptual background: Aggression is a multi-faceted social behavior [1,85], which can be regarded as a downstream dysfunctional behavior resulting from core symptoms of BPD [31]. Recently, we proposed a model of aggression in BPD from the perspective of biobehavioral dimensions resp. mechanisms [86] suggesting impulsivity and affect dysregulation to be particularly important biobehavioral dimensions underlying aggressive behavior in BPD.

Within this framework, the findings reviewed above may point to a particular importance of the biobehavioral dimension of impulsivity for male BPD patients’ aggressiveness. Most of the neurobiological alterations that have been reported in male but not in female BPD patients in comparison to gender-matched healthy volunteers, namely the volume loss of the anterior cingulate cortex [58,87], the reduced serotonergic responsivity [88] as well as the structural and functional abnormalities of striatal brain structures [89], have been related to reduced impulse control. Additionally, male compared to female BPD patients scored higher on impulsivity [90] (cf. [91]) and explosive temperaments [92]. In line with this are findings from the general population, where the male-over-female preponderance in aggression has also been traced back to higher impulsivity [93] and/or reduced behavioral inhibition [15].

As mentioned above, specific alterations in female BPD patients are rare. Hypothesizing that dysfunctions of the amygdala might be more prominent in female than in male patients, one could speculate that affective dysregulation [94] may play a specific role in female BPD patients’ aggressiveness.

The following limitations should be noted. First, studies analyzing mixed-gender samples are scarce. To exclude a potential confounding influence of gender, the majority of the neurobiological mixed-gender studies reported here compared the patients with gender-matched healthy controls, e.g. male BPD patients with male controls. To our knowledge, only two neuroimaging studies [28,63] have directly compared male with female patients. Although the approach to compare BPD patients with controls of the same gender primarily seems reasonable to us, it does not enable an understanding to be gained of whether and how the BPD diagnosis interacts with gender. Future studies including sufficiently large gender-mixed samples, which in addition to gender-matched comparisons also compare male and female BPD patients, are therefore requested in order to investigate whether BPD psychopathology impinges differently upon women and men.

Comparing male directly with female BPD patients may also be of importance in terms of gender-specific treatments. For instance, psychopharmacological substances targeting the serotonin system may lead to different effects in male versus female BPD patients. However, the – at least to our knowledge – only study to date to have used the serotonin reuptake inhibitor fluoxetine to treat male and female patients with intermittent explosive disorder, and in part comorbid BPD, did not find a significant drug x gender interaction [95]. Psychotherapeutic treatments might profit from different treatment foci, which consider gender-specific differences in mechanisms underlying aggression in BPD. For instance, male BPD patients may particularly benefit from interventions aiming to increase impulse control, as, e.g., implemented in the Dialectical Behavioral Therapy [96].

Upcoming studies may also benefit from considering results of neurobiological gender differences found in precursor syndromes associated with aggression and BPD, such as conduct disorder and attention deficit hyperactivity disorder. Male patients with conduct disorder exhibited larger volumes of the anterior insula than their female counterparts [97]. Men affected with attention deficit hyperactivity disorder demonstrated reduced activation during a working memory task in frontal, temporal, subcortical, occipital and cerebellar regions relative to healthy males, whereas affected and healthy females demonstrated equivalent activations [98]. These findings underline the importance of investigating similar tasks in BPD patients.

Second, results in the general population emphasize the importance of considering different contributions to gender differences depending on the form of aggression under focus [14,15]. Understanding aggression as a complex and heterogeneous social behavior, and thoroughly differentiating between, e.g., verbal and physical forms of aggression could help to reduce inconsistencies.

Third, the potential confounding effect of comorbidities needs to be stressed. As pointed out throughout the article, male BPD patients frequently suffer from higher prevalence rates of aggression-predisposing disorders such as ASPD. Future studies should control this influence, e.g. by analyzing matched samples. The confounding influence of comorbidities also implies the use of a dimensional rather than a categorical approach to BPD psychopathology, as has been proposed by the alternative DSM-5 model of BPD [2] and recent research on BPD psychopathology [86].

### Funding

This work was supported by two grants of the German Research Foundation to S. C. H. on borderline personality disorder (Clinical Research Foundation on Mechanisms of Disturbed Emotion Processing in Borderline Personality Disorder, www.kfo256.de; Schmahl et al., 2014: He 2660/12-1; He 2660/7-2) as well as financial support by the German Research Foundation and Ruprecht-Karls-Universität Heidelberg within the funding program Open Access Publishing.
